# Setting a research agenda for progressive multiple sclerosis: The International Collaborative on Progressive MS

**DOI:** 10.1177/1352458512458169

**Published:** 2012-11

**Authors:** Robert J Fox, Alan Thompson, David Baker, Peer Baneke, Doug Brown, Paul Browne, Dhia Chandraratna, Olga Ciccarelli, Timothy Coetzee, Giancarlo Comi, Anthony Feinstein, Raj Kapoor, Karen Lee, Marco Salvetti, Kersten Sharrock, Ahmed Toosy, Paola Zaratin, Kim Zuidwijk

**Affiliations:** 1 Mellen Center for Multiple Sclerosis, Neurological Institute, and Lerner College of Medicine, Cleveland Clinic, Cleveland, OH, USA; 2 Department of Brain Repair and Rehabilitation, University College London Institute of Neurology, Faculty of Brain Sciences, London, UK; 3 Centre for Neuroscience and Trauma, Blizard Institute, Barts and The London School of Medicine and Dentistry, Queen Mary University of London, UK; 4 Multiple Sclerosis International Federation, London, UK; 5 Multiple Sclerosis Society, London, UK; 6 National MS Society, New York, NY, USA; 7 Department of Neurology, Scientific Institute San Raffaele, Milan, Italy; 8 Department of Psychiatry, University of Toronto, Canada; 9 National Hospital for Neurology and Neurosurgery, London, UK; 10 MS Society of Canada, Toronto, ON, Canada; 11 Center for Neurology and Experimental Therapies, Sapienza University, Rome, Italy; 12 Fast Forward, LLC, New York, NY, USA; 13 Italian MS Society, Genoa, Italy; 14 MS Research Foundation, Voorschoten, The Netherlands

**Keywords:** multiple sclerosis, progressive multiple sclerosis, neuroprotection, rehabilitation, research agenda

## Abstract

Despite significant progress in the development of therapies for relapsing MS, progressive MS remains comparatively disappointing. Our objective, in this paper, is to review the current challenges in developing therapies for progressive MS and identify key priority areas for research. A collaborative was convened by volunteer and staff leaders from several MS societies with the mission to expedite the development of effective disease-modifying and symptom management therapies for progressive forms of multiple sclerosis. Through a series of scientific and strategic planning meetings, the collaborative identified and developed new perspectives on five key priority areas for research: experimental models, identification and validation of targets and repurposing opportunities, proof-of-concept clinical trial strategies, clinical outcome measures, and symptom management and rehabilitation. Our conclusions, tackling the impediments in developing therapies for progressive MS will require an integrated, multi-disciplinary approach to enable effective translation of research into therapies for progressive MS. Engagement of the MS research community through an international effort is needed to address and fund these research priorities with the ultimate goal of expediting the development of disease-modifying and symptom-relief treatments for progressive MS.

The last two decades have seen dramatic progress in relapsing–remitting multiple sclerosis (RRMS). Experimental autoimmune encephalomyelitis (EAE) and other animal models have provided insights into the pathophysiology of central nervous system inflammation and demyelination. Clinical diagnostic criteria have been refined, and biomarkers are being developed that predict future disease activity and disability. A clear pathway has emerged for developing RRMS therapies: studies in experimental models, Phase I safety studies, Phase II trials with active MRI lesions as the primary outcome, and finally Phase III trials using relapses and sustained progression of disability as primary outcome. Eight disease-modifying therapies have received regulatory approval for RRMS and several more are in late-stage clinical development and could receive regulatory approval shortly.

For all the success in developing treatments for RRMS, the story in progressive MS is comparatively disappointing and more challenging. Even the definition of progressive MS has been elusive. At the clinical level, progressive MS is defined as the gradual progression of clinical disability in a patient either with a preceding relapsing course (secondary progressive MS, SPMS) or without a preceding relapsing course (primary progressive MS, PPMS).^1^ There may be superimposed evidence of overt inflammation, but frequent relapses and many new lesions on MRI are more suggestive of RRMS. At the imaging level, progressive MS is the gradual accumulation of imaging abnormalities. At the pathology level, progressive MS is the abnormal processes present in neurons or glial cells that lead to irreversible injury that causes clinical disability progression. An inherent difficulty in studying progressive MS is the indistinct overlap with RRMS, with the pathologic origins of progressive MS probably developing much earlier than its clinical manifestations. Here, PPMS and SPMS are grouped together, since they share many similarities – clinically, pathologically, and particularly as revealed by imaging technology.^2^

Animal models such as EAE provide only limited insight into the pathophysiology of progressive MS. New MRI lesions are only occasionally seen in progressive MS, resulting in uncertainty as to which imaging or other biomarker should be employed in Phase II proof-of-concept clinical trials. The clinical metrics used in RRMS have unclear sensitivity in progressive MS, limiting their utility. Mechanisms for identifying candidate therapies among existing therapies are not well defined.

Clinical trials of anti-inflammatory therapies in progressive MS have been generally negative or inconsistent. Immunosuppressive and immunomodulating drugs such as cladribine, azathioprine, and cyclophosphamide have shown no evidence for efficacy in SPMS and PPMS. Only mitoxantrone has been approved for SPMS in some countries, and this treatment has a serious adverse effect profile. Finally, early attempts to approach progressive MS with putative neuroprotective therapies have failed, as seen in the recent trial of lamotrigine.^3^

Given the challenges presented by progressive MS, a collaborative was convened by volunteer and staff leaders from several MS societies ‘to expedite the development of effective disease-modifying and symptom management therapies for progressive forms of multiple sclerosis’. Through a series of scientific and strategic planning meetings, five key priority areas for research were identified (Table 1). These areas represent opportunities where concerted research efforts would provide significant impact in overcoming the current barriers in developing effective treatments for progressive MS and provide a clear roadmap for the future.

**Table 1.** Five key research priorities for progressive MS.

- Experimental Models
- Identification and Validation of Targets and Repurposing Opportunities
- Proof-of-Concept Clinical Trial Strategies
- Clinical Outcome Measures
- Symptom Management and Rehabilitation

## Experimental models

Experimental models for MS have provided important insights into disease pathogenesis and potential therapies.^4^ Neurotoxicity models inform neuroprotection strategies that may prevent neurodegeneration in MS.^4^ The three most commonly studied animal models in MS are: EAE, virally induced demyelinating disease models and toxin-induced models of demyelination.^5^ Despite their extensive use, the clinical course, immunology and neuropathology of these models reflect only part of the pathophysiological spectrum of human MS.^4,6^ Therefore, direct extrapolation of results obtained in these models to MS is often tenuous, and the effect of therapeutic interventions in animals must be interpreted with care.^7^ In addition, most of the current models follow a monophasic episode of inflammation and therefore predominantly mirror only the impact of acute inflammation and neuronal injury seen in RRMS.^4,6^

Several animal models claim to represent human progressive MS,^8^ but few offer compelling evidence.^6^ The chronicity of these models is usually short and the composition of lesions different from MS.^6^ Furthermore, most of these models do not reflect the irreversible deficits characterizing progressive MS.^9^

Therefore, there is an urgent need for better animal models that reproduce the key clinical and pathological features of SPMS and PPMS. Such models should include the role of CD8-positive T cells and notably macrophages, which comprise a major component in progressive MS lesions. Ectopic B cell follicles should be studied as an alternative disease-related mechanism. Additional models are also needed that demonstrate robust chronic demyelination and neurodegeneration, such as an autoimmune-independent, inflammatory glial cell-associated neurodegeneration, which more accurately reflects progressive MS. In addition to animal models, brain-slice cultures that demonstrate demyelination and neurodegeneration may be informative, especially if they can be adapted to use human post-mortem tissue where MS pathology can be directly examined.

## Identification and validation of targets and repurposing opportunities

While our understanding of the pathophysiology of progressive MS is yielding knowledge which can be applied to the development of new treatments, identifying and validating targets for use in drug discovery for progressive MS remains a significant challenge. Helpful insights may come from genome-wide association studies (GWAS). Since the first non-MHC susceptibility locus (IL2RA) was identified,^10^ the list of MS risk loci is growing, with around 50 loci identified to date.^11^ However, no individual gene variant has been identified as the ideal therapeutic target and, even when considering all disease-associated variants together, risk loci explain only a modest fraction of disease heritability.^12^ Notwithstanding these limitations, the available results may suffice for computational and systems biology analyses to provide novel insights into biological pathways involved in disease pathogenesis. Indeed, computational biology is shifting from diagrammatic representation of pathways to mathematical models. These techniques hold promise to provide the tools for interpreting genetic data across different knowledge domains.^13^ Furthermore, systematic assessments of the functional consequences of the associated variants are underway. Together, the results of these studies could help prioritize putative therapeutic targets and steer the development of new compounds.

In addition, methods have recently been developed to select old drugs for new targets. Historically, repositioning of a compound for a new indication has been a chance occurrence, driven by observations of unforeseen favorable effects. Now there is intense research on how to systematically infer new therapeutic targets for drugs that have already been registered for human use. Side-effect or chemical similarities between drugs and ligand sets are examples of this new strategy.^14,15^ These data may be further refined through screenings of registered drugs on cellular and animal models of MS.

Superimposing biologically relevant pathways identified through GWAS studies to ‘catalogues’ of phenotypic effects of registered drugs may shorten the process of identifying therapies with potential efficacy in progressive MS. This strategy is safe for patients, since repurposed drugs usually come with years of post-marketing experience in other diseases. This strategy is also cost-effective, since it streamlines preclinical and early-stage clinical studies. Indeed, the latest GWAS data did not show substantial differences at susceptibility loci between relapsing and primary progressive forms of MS, suggesting relevance of GWAS results to progressive MS.^11^

The therapeutic opportunities that come from GWAS, computational biology and systematic reassessment of the effects of pharmaceutical compounds need further development to achieve their potential application to progressive MS. Intellectual property and the possibility of re-evaluating compounds that have not made it through the approval process are among the issues that, if properly addressed, may help accelerate the development of effective therapies for progressive MS.^16^

## Proof-of-concept clinical trial strategies

Agents for which there is promising preclinical data need to be developed through early phase clinical trials for evidence of safety and therapeutic benefit. Phase II trials generally rely on biomarkers that are more sensitive to therapeutic effects than clinical measures. Biomarker outcomes enable Phase II trials to be shorter and have a smaller sample size than Phase III trials.

Valid surrogate biomarkers need to predict clinical outcomes. Phase II trials in RRMS have advanced because lesion activity on MRI is an accepted biomarker of clinical relapse rate.^17^ In contrast, no comparable measure has been identified in progressive MS. There are no agreed imaging markers of neurodegenerative processes such as energy failure, ionic imbalances and loss of neuronal integrity. The problem is compounded by limitations of clinical measures of disease progression against which any biomarker might be validated.

At present, promising imaging metrics include cerebral and spinal cord atrophy, lesion T1 hypointensity, magnetization transfer ratio to assess lesion microstructure, and optical coherence tomography to measure axonal degeneration in the retina.^18^ There is enough longitudinal data to enable sample sizes to be calculated for most of these techniques for proof-of-concept trials, but their sensitivity to change and responsiveness to treatment is not well understood. Newer techniques that assess tissue microstructure are also candidate metrics, including diffusion tensor imaging^19^ in addition to methods that can derive axonal density and radius. Techniques that examine earlier events in the injury pathway include sodium imaging^20^ and measurements of metabolic markers, including the neuronal/mitochondrial marker N-acetylaspartate.^21^ These techniques could be complemented and extended by positron emission tomography (PET).^22^ Little is known about the sensitivity, responsiveness and predictive power of most of these imaging techniques and their limited availability may restrict their widespread use.

A number of tissue fluid biomarkers have been studied, mostly to assess immunological activity. Markers of specific injury mechanisms are also emerging, including chemokines associated with intrathecal B lymphocyte activity that might drive cortical injury,^23^ nitric oxide metabolites,^24^ and neurofilaments released by damaged axons.^25^ A significant drawback to widespread application of these biomarkers is their typical measurement from cerebrospinal fluid, which is not easily accessible. Nonetheless, cerebrospinal fluid is increasingly incorporated into designs of progressive MS trials, and efforts to identify biomarkers in plasma and serum are underway.

Better biomarkers would power smaller and shorter trials. More flexible trial designs are also being examined to achieve the same aim. These include modified entry criteria to enrich trials for patients more likely to progress. Adaptive designs such as those used in cancer trials could make use of prognostic biomarkers to stratify an outcome analysis that is sensitive to subpopulation treatment effects,^26^ and could employ an interim futility analysis to exclude non-effective agents.^27^

These considerations suggest that proof-of-concept clinical trial strategies are likely to evolve significantly if (1) biomarkers can be identified and validated that measure important events in the neuronal injury pathway, are reliable, easily implemented, dynamic over time, and correlate with disability, and (2) if trial designs can be developed which further minimize trial size and duration. Importantly, these innovations will need sufficient community consensus to be accepted by regulatory authorities.

## Clinical outcome measures

A critical aspect in the development of therapies is a measurement tool of therapeutic efficacy. The ideal measurement tool is precise, reproducible, broad-based in its assessment, sensitive to change over time, and predictive of future change. The evaluation of MS therapies in RRMS was greatly assisted by clear definitions and objective measurement of clinical relapses. Establishing outcome measures for progressive MS has been more difficult. This difficulty arises from the varied manifestations of progressive MS (motor, sensory, coordination, cognitive, etc.), their slow rate of evolution, and difficulties in their quantitative measurement. There are two main pathways to solving this challenge: refinement of existing outcome measures and development of new outcome measures.

Of the existing measures, The Kurtzke Expanded Disability Status Scale (EDSS)^28^ is the most common disability measure in MS trials. However, EDSS is an inherently subjective assessment by a neurologist, has poor intra- and inter-rater reliability,^29^ and has poor precision. Refinements to the EDSS would likely improve its performance, although many of its shortcomings are inherent to the tool and so are insurmountable.

The Multiple Sclerosis Functional Composite (MSFC) is the outcome of an international panel charged with replacing the EDSS.^30^ Advantages to the MSFC include its dynamic assessment of different functions relevant to MS (ambulation, arm function, and cognition) and improved statistical performance. Since the introduction of the MSFC, many validation studies have shown its clinical correlations and predictive capacity. Despite these apparent advantages, the MSFC has not always been more sensitive than the EDSS in clinical trials. Equally important, the MSFC has not yet been accepted by regulators as an alternative to EDSS. As with EDSS, further refinement of the MSFC may improve its sensitivity, reliability, and responsiveness, although some shortcomings cannot be overcome.^31^

Important goals of MS therapies are to reduce symptom severity, improve function, and enhance quality of life. These are best evaluated through patient-reported outcome measures (PROMs), including global assessments of daily function and health-related quality of life (HRQL) measures.^32^ Regulatory interest in PROMs is growing, with guidelines emerging regarding the integration of PROMs into clinical trials.^33^ Efforts to improve clinical disability assessment in MS are already underway.^31^

## Symptom management and rehabilitation

MS results in a diversity of symptoms, bringing increasing physical, psychological and emotional burden, particularly in the progressive stage of the condition. In spite of the introduction of effective disease-modifying treatments, symptom management and rehabilitation remain essential components of MS therapy, helping to alleviate the impact of disability and improve quality of life. Surprisingly, the rationale for specific pharmacological treatments for symptoms is frequently based on few trials with small patient numbers, often underpowered and unblinded.^34^ Recently, these shortcomings have begun to be addressed with a few well conducted studies, such as using cannabinoids^35^ and fampridine^36^ for motor symptoms. While cognitive deficits can now be clearly defined, fatigue remains more difficult to evaluate in trials because (a) less is known about the pathophysiology and (b) it may be influenced by psychiatric factors, making quantitative characterization problematic and often confounded. Depression and anxiety may respond well to either pharmacologic or cognitive behavioral treatment.^37–39^ Symptom management in MS can be advanced in a number of ways. First, there should be targeted research to improve our understanding of the pathological mechanisms leading to symptom-related disability. This knowledge will allow more focused translational steps towards developing symptomatic therapies. Secondly, potential treatments should be assessed in rigorous, well designed trials that are sufficiently powered to establish beneficial effects, the optimum dosage, and short- and long-term side effects. Potential symptom interactions and confounding factors should be accounted for in the trial design. Ideally, studies should incorporate PROMs, surrogate pathological markers related to the particular symptom under study and an assessment of cost-effectiveness. Third, further development of reproducible and responsive measures for different symptoms is needed.

Symptomatic treatment is normally part of a multidisciplinary patient-centered approach that may involve rehabilitation. The relationship between neuroplasticity and rehabilitation is a critically important area for further research. Functional brain reorganization is well described in MS, showing increasing activation extent and the recruitment of additional areas, and hinting at the prospect of compensatory strategies.^40,41^ Some motor networks may be altered by training^42^ but there is a need to investigate if enhancing network plasticity may improve the outcomes of rehabilitation. Combining functional and structural imaging with cognitive rehabilitation may help develop treatments for cognitive impairment.^43^ Finally, applying brain–computer interface technology in patients with advanced MS may allow greater motor independence, communication and environmental control.^44^

## Conclusions and future directions

Despite great progress in relapsing MS, much work is needed to achieve similar successes for progressive MS. There are a number of key areas of unmet need which are blocking treatment development in progressive MS. Although the international scientific community has made progress in some of these areas (Table 2), there have not been commensurate gains in progressive MS treatments. Tackling these issues will require an integrated, multi-disciplinary approach to enable effective translation of research into therapies. To this end, the International Progressive MS Collaborative is committed to engaging the MS research community through an international effort to fund a spectrum of research activities relevant to progressive MS with the ultimate goal of expediting the development of disease-modifying and symptom-relief treatments for progressive MS.

**Table 2.** Examples of ongoing projects in progressive MS.

- Clinical trials, including Phase II and Phase III trials
❍ fingolimod, rituximab, several stem cell studies, and other potential disease-modifying therapies
❍ phenytoin, amiloride, cannabinoids, and other symptomatic therapies
- MS phenotypic project (NMSS/ECTRIMS Clinical Trials Committee)
- Multiple Sclerosis Functional Composite Study Group
- Risk Factors for MS Progression Project
- Pathobiology of MS: complex interplay between degeneration and inflammation (Multiple Sclerosis Scientific Research Foundation Multi-Center Collaborative Grant)
- UK MS Clinical Trials Network
- Multiple Sclerosis Functional Composite Task Force, National MS Society

To address these five challenging areas, which currently impede the treatment of progressive MS, the International Progressive MS Collaborative commissioned five working groups, comprised of international experts, to identify specific strategies and potential lines of research that would overcome the barriers and realize the opportunities within each area. Following an international meeting in early 2013, we anticipate that a call will be issued to address these opportunities. Potential sources of funding for this call include the existing research funding mechanisms of the member organizations of the International Progressive MS Collaborative as well as other partners (e.g., government, industry). In addition, there will be an international fundraising effort led by the Multiple Sclerosis International Federation and financial support will be solicited from diverse channels around the world, including foundations, government, corporate, and private funding organizations.

Fostering global collaboration by the MS research community is a bold ambition, and potentially fraught with many challenges. Fortunately, the opportunities have never been as favorable as they are today, with unprecedented data on disease etiology, pathophysiology, and disease course. Furthermore, we can look to other diseases for inspiration. Collaborative efforts like the Forum for Collaborative HIV Research, the Alzheimer’s Disease Neuroimaging Initiative, and the Innovative Medicines Initiative, provide powerful examples of how collaboration can accelerate research among a diverse group of stakeholders. While the collaborative efforts in progressive MS will almost certainly differ from those in other fields, the time is right for concerted action.
